# Data on calcium increases depending on stretch in dystrophic cardiomyocytes

**DOI:** 10.1016/j.dib.2016.08.011

**Published:** 2016-08-10

**Authors:** E. Aguettaz, J.J. Lopez, A. Krzesiak, B. Constantin, C. Cognard, S. Sebille

**Affiliations:** a Laboratoire de Signalisation et Transports Ioniques Membranaires (STIM), Equipe Transferts Ioniques et Rythmicité Cardiaque (TIRC), Université de Poitiers, 86073 Poitiers Cedex 9, France; b Laboratoire de Signalisation et Transports Ioniques Membranaires (STIM), Equipe Calcium et Microenvironnement des Cellules Souches (CMCS), Université de Poitiers, 86073 Poitiers Cedex 9, France

**Keywords:** Calcium, TRPs channels, Stretch, Cardiomyocytes, Dystrophic

## Abstract

In this data article, intracellular Ca^2+^ concentration ([Ca^2+^]_i_) was measured in isolated ventricular Wild Type (WT) and *mdx* cardiomyocytes in two different conditions: at rest and during the application of an axial stretch. Using a carbon microfibers technique, axial stretch was applied to mimic effects of physiological conditions of ventricular filling. A study of cation entry with the same experimental model and the manganese quenching method reported (i) a constitutive cation entry in *mdx* cardiomyocytes and (ii) the involvement of TRPV2 channels in axial-stretch dependant cation entry, “Axial stretch-dependent cation entry in dystrophic cardiomyopathy: involvement of several TRPs channels” (Aguettaz et al., 2016) [1].

Here, the Ca^2+^ dye fluo-8 was used for [Ca^2+^]_i_ measurement, in both resting and stretching conditions, using a perfusion protocol starting initially with a calcium free Tyrode solution followed by the perfusion of 1.8 mM Ca^2+^ Tyrode solution. The variation of [Ca^2+^]_i_ was found higher in *mdx* cardiomyocytes.

**Specifications Table**

**Table:** 

Subject area	*Biology*
More specific subject area	*Calcium regulation in cardiomyopathy*
Type of data	*Figures*
How data was acquired	*Confocal microscopy, BIORAD 1024*
Data format	*Raw, analysed*
Experimental factors	*Isolated mouse cardiomyocytes were axial stretched*
Experimental features	*Intracellular calcium changes were recorded*
Data source location	*Poitiers, France*
Data accessibility	*Data is within this article*

**Value of the data**

• Protocol used in these experiments could activate Store-operated calcium channels (SOCs) and stretch-activated calcium channels (SACs).
• This protocol can be used to investigate stretch-dependent calcium increases in cardiomyocytes and other cells.
• The effects of SOCs and SACs inhibitors and activators may help to understand stretch-dependent calcium increases.

## 1 Data

**Fig. 1:**
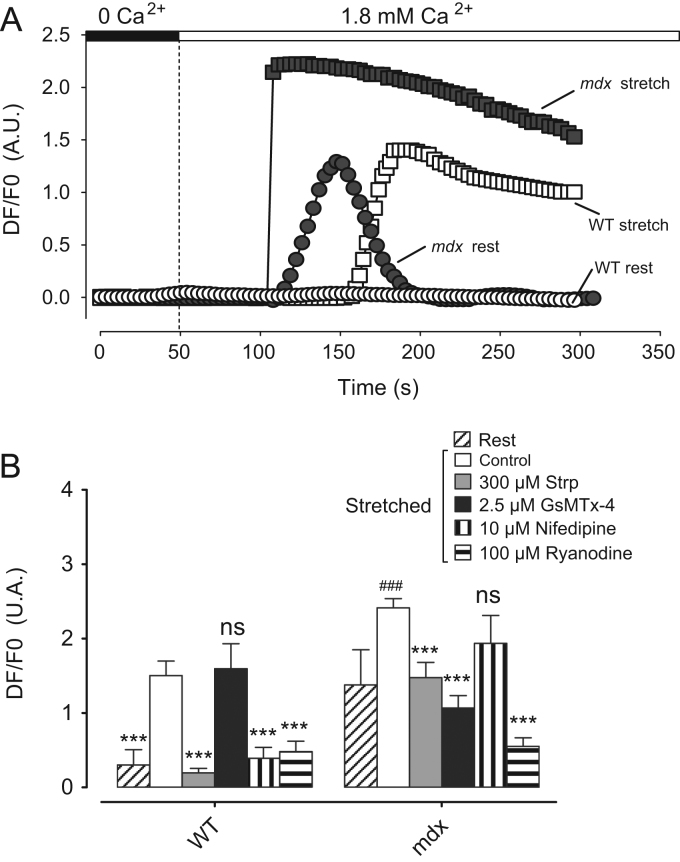
(A) Representative recordings relative to fluo-8 fluorescence (expressed as DF/F0 (A. U.)) in resting WT (open circles) and *mdx* (filled circles) and in stretched WT (open squares) and *mdx* (filled squares) during a superfusion protocol starting initially with a calcium free Tyrode solution followed by the superfusion of 1.8 mM Ca^2+^ Tyrode solution. (B) Maximal amplitude of fluo-8 fluorescence intensity in WT and *mdx* cardiomyocytes maintained in stretched condition with SACs inhibitors: cells were incubated with 300 µM streptomycin (Strp, gray bars) or 2.5 µM GsMTx-4 (Black bars) for SACs inhibition and with 10 µM nifedipine (vertical hatching) or 100 µM ryanodine (horizontal hatching) for EC coupling inhibition. Open bars represent the control. Declined hatching represents rest (non-stretched). Measurements are represented as mean normalized fluo 8 fluorescence intensity±SEM. Rest (WT: *n*=10 cells, *N*=4 hearts; *mdx*: *n*=12 cells, *N*=4 hearts). stretch control (WT: *n*=12 cells, *N*=4 hearts; *mdx*: *n*=12 cells, *N*=4 hearts). Strp (WT: *n*=7 cells, *N*=3 hearts; *mdx*: *n*=6 cells, *N*=3 hearts). Rest (WT: *n*=10 cells, *N*=4 hearts; *mdx*: *n*=12 cells, *N*=4 hearts). GsMTx-4 (WT: *n*=5 cells, *N*=3 hearts; *mdx*: *n*=5 cells, *N*=3 hearts). Nifedipine (WT: *n*=5 cells, *N*=4 hearts; *mdx*: *n*=5 cells, *N*=3 hearts). Ryanodine (WT: *n*=9 cells, *N*=4 hearts; *mdx*: *n*=7 cells, *N*=4 hearts). * Symbol represents the statistical difference with control, ***P<0.001; ns, not significant. # Symbol represents the statistical difference between WT and *mdx* in stretched conditions. ^###^*P*<0.001.

**Fig. 2:**
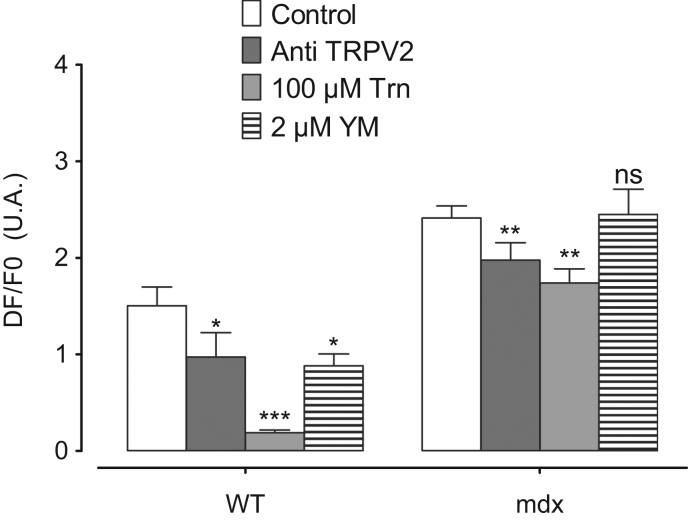
Maximal amplitude of fluo 8 fluorescence in WT and *mdx* cardiomyocytes maintained in stretching condition incubated with TRPs inhibitors. Cells were incubated with TRPs blockers: antibody against an extracellular epitope of TRPV2 (Anti-TRPV2 : dark gray bars), 100 µM tranilast (horizontal hatching) and YM-48483, inhibitor of TRPCs channels in stretched WT and *mdx* cardiomyocytes. Open bars represent the control. Measurements are represented as mean normalized fluo-8 fluorescence intensity±SEM. Control (WT: *n*=10 cells, *N*=4 hearts; *mdx*: *n*=12 cells, *N*=5 hearts). Anti-TRPV2 (WT: *n*=6 cells, *N*=4 hearts; *mdx*: *n*=6 cells, *N*=4 hearts). Trn (WT: *n*=6 cells, *N*=3 hearts; *mdx*: *n*=7 cells, *N*=4 hearts). YM-48483 (WT: *n*=8 cells, *N*=4 hearts; *mdx*: *n*=9 cells, *N*=4 hearts). * Symbol represents the statistical difference with control *P<0.01; **P<0.005; ***P<0.001; and ns, no significant.

**Fig. 3:**
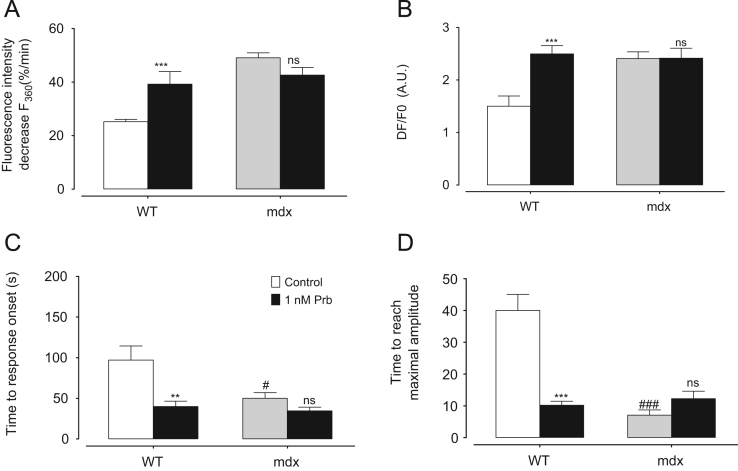
Maximal amplitude of fluo 8 fluorescence in WT and *mdx* cardiomyocytes maintained in stretching condition incubated with probenecid. Cells were incubated with 1 nM probenecid (Prb, black bars). Open bars represent the control and clear gray bars *mdx* cardiomyocytes in resting conditions. Measurements are represented as mean normalized fluo-8 fluorescence intensity±SEM. (C) Time between the beginning of the 1.8 mM Ca^2+^ perfusion and the start of the normalized fluo-8 fluorescence intensity increases. Measurements are represented as mean of the time to response onset (expressed in seconds)±SEM. (D) Time between the start of the normalized fluo-8 fluorescence intensity increase and maximal amplitude. Measurements are represented as mean of the time to reach maximal amplitude of fluorescence signal (expressed in seconds)±SEM. Control (WT: *n*=8 cells, *N*=3 hearts; *mdx*: *n*=12 cells, *N*=4 hearts). Prb (WT: *n*=12 cells, *N*=4 hearts; *mdx*: *n*=8 cells, *N*=4 hearts). * Symbol represents the statistical differences with control. *P<0.01; **P<0.005; ***P<0.001; and ns, no significant. # Symbol represents the statistical difference between WT and *mdx* in stretched conditions. ^#^*P*<0.01; and ^###^*P*<0.001. ns, no significant.

These data mainly focus on describing intracellular calcium concentration ([Ca^2+^]_i_) measured in Wild Type (WT) and *mdx* cardiomyocytes in two different conditions: at rest and during the application of an axial stretch. Calcium measurement was performed using the Ca^2+^ dye fluo-8 and during a perfusion protocol starting initially with a calcium free Tyrode solution followed by the perfusion of 1.8 mM Ca^2+^ Tyrode solution. In these conditions, [Ca^2+^]_i_ kinetics are described (Fig. 1A) and amplitudes are compared in the presence of SACs inhibitors (Fig. 1B). The effect of TRPV2 inhibitors (Fig. 2) and Probenecid (Fig. 3) is also described.

## 2 Experimental design, materials and methods

### 2.1 Cells isolation, mechanical stimulation and Mn^2+^-quenching experiments are described in [1]

#### 2.1.1 Solutions and chemicals

Before experiments, cells were stored in culture medium containing Dulbecco׳s modified Eagle׳s medium (DMEM – Lonza: 12-604F), complemented with 10 µg/mL insulin, 10 µg/mL gentamycin, 4 mM NaHCO_3_, 10 mM Hepes, 0.2% BSA and 12.5 µM blebbistatin (all from Sigma). During experiments, cardiomyocytes were superfused with Tyrode solution containing 140 mM NaCl, 5.4 mM KCl, 1.8 mM CaCl_2_, 1.8 mM MgCl_2_, 10 mM Hepes and 11 mM glucose or with calcium-free Tyrode solution containing 140 mM NaCl, 5.4 mM KCl, 1.8 mM MgCl_2_, 10 mM Hepes, 11 mM glucose, 1 g/L BSA and 20 mM Taurine.

Tranilast (Trn) was purchased from Calbiochem (53902-12-8), GsMTx-4 from Abcam (ab141871), probenecid (Prb, P8761), Streptomycin sulfate (Strp, s9137) and 4-methyl-4′-[3.5-bis(trifluoromethyl)-1H-pyrazol-1-yl]-1.2.3-thiadiazole-5-carboxanilide (YM-58483, y4895) from Sigma, ryanodine from Merck (15662-33-6), fura-2-AM (108964-32-5) and fluo-8-AM (sc-362561) from Santa-Cruz.

#### 2.1.2 Intracellular calcium measurements using confocal microscopy

Cells were loaded at room temperature during 20 min with the single-wavelength calcium sensitive probe fluo-8-AM (6 µM) in Ca^2+^-free Tyrode solution. Cells were stretched at 10% of the initial sarcomere length before recording fluorescence.

Fluorescence images were recorded by Confocal Laser Scanning Microscopy (CLSM) using a Bio-Rad MRC 1024 (Bio-Rad Laboratories) equipped with a 15 mW Ar–Kr gas laser. Confocal unit was attached to an inverted microscope (Olympus IX70) with a 60× water objective and fluorescence signal collection was performed through the control software Lasersharp 3.2 (Bio-Rad Laboratories). Fluorophore was excited with the 488 nm line and the emitted fluorescence was detected at 522 nm (green fluorescence). For analysis, fluorescence was normalized (DF/F0) and calculated through IDL software routines [2].

#### 2.1.3 Statistical analysis

Data are presented as means±SEM, *n* is the number of cells. Differences were tested with *t*-test. *P*<0.05 indicates a statistical significant difference.

## References

[1] Aguettaz E., Lopez J.J., Krezsiak A., Lipskaia L., Adnot S., Hajjar R.J., Cognard C., Constantin B., Sebille S. (2016). Axial stretch-dependent cation entry in dystrophic cardiomyopathy: involvement of several TRPs channels. Cell Calcium.

[2] Mondin L., Balghi H., Constantin B., Cognard C., Sebille S. (2009). Negative modulation of inositol 1,4,5-trisphosphate type 1 receptor expression prevents dystrophin-deficient muscle cells death. Am. J Physiol. Cell Physiol..

